# Incidental Finding of Bladder Herniation Through the Obturator Canal: A Cadaveric Observation

**DOI:** 10.7759/cureus.80884

**Published:** 2025-03-20

**Authors:** Jessica Scott, Trevor Jackson, Pooja Patel, Shreya Bhatt, Olga Avilova, Mohammadali M Shoja

**Affiliations:** 1 Medical Education, Nova Southeastern University Dr. Kiran C. Patel College of Allopathic Medicine, Fort Lauderdale, USA; 2 Medical Education, Nova Southeastern University Dr. Kiran C. Patel College of Osteopathic Medicine, Fort Lauderdale, USA

**Keywords:** anatomical pathological condition, chronicity, incidental findings, obturator hernias, postmortem diagnosis

## Abstract

Obturator bladder hernia is an exceedingly rare condition, presenting with groin or pelvic pain or nonspecific bladder symptoms. We report an incidental finding of a chronic, long-standing obturator bladder hernia during the routine dissection of a donor with a thin body habitus. The hernia presented as a pedunculated extension of the anterolateral upper aspect of the bladder, traversing the right obturator foramen and flattening externally between the external obturator and pectineus muscles. Focal adhesions at the obturator foramen and dilation of the obturator canal suggested chronicity. Although incidentally discovered, the anatomical location of this hernia could potentially have caused obturator nerve compression. Our observations suggest that the true incidence of insidious obturator hernias may be underreported, emphasizing the need for increased clinical vigilance and routine pelvic imaging. This case further highlights the importance of recognizing chronic obturator hernias, particularly in elderly, frail individuals, as they may go undetected until incidental findings or post-mortem examinations.

## Introduction

The obturator canal is an osseofibrous canal situated between the origins of the internal and external obturator muscles, bounded superiorly by the superior pubic ramus and medially, inferiorly, and laterally by the obturator membrane and the obturator muscles [1]. This canal connects the pelvis to the medial compartment of the thigh and generally measures between 0.2 and 0.5 cm in width and 3 cm in length [2]. The obturator nerves and vessels traversing the canal are enveloped in adipose tissue. In patients with malnutrition, depletion of this adipose layer increases the risk of herniation of intra-abdominal contents through the obturator foramen [3]. An obturator hernia is a rare condition that frequently involves small intestines, although reports have documented herniation of other organs, such as the omentum, large intestine, appendix, bladder, ureter, ovary, and fallopian tube [2,4,5]. An obturator bladder hernia is an exceedingly rare condition, with only nine cases documented in the literature. Among these, only three cases have been identified incidentally [5-7]. In this report, we describe an incidental finding of an obturator bladder hernia during the dissection of a donor cadaver as part of a medical education curriculum. The anatomy of this hernia and its clinical relevance are analyzed in the context of our dissection findings.

## Case presentation

**Figure 1:**
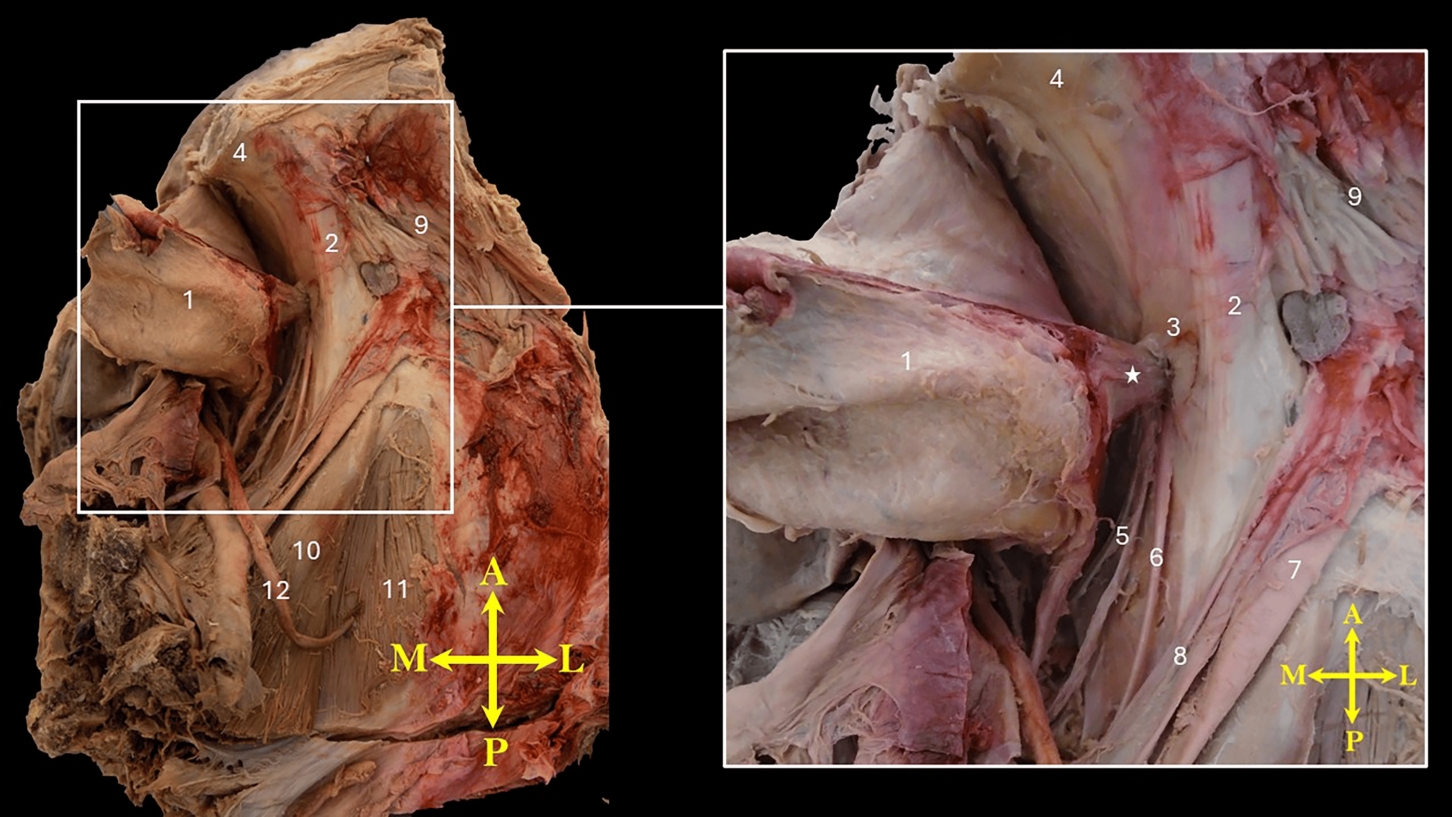
Superior view of the isolated right hemipelvis demonstrating an extension of the superolateral corner of the urinary bladder (from the junction of its dome and lateral surface) herniating into the obturator foramen. The neck of the hernia is marked by an asterisk (*). 1, urinary bladder; 2, superior pubic ramus; 3, obturator foramen; 4, posterior surface of the body of the pubis; 5, obturator vessels; 6, obturator nerve; 7, external iliac artery; 8, external iliac vein; 9, pectineus muscle; 10, psoas major muscle; 11, iliacus muscle; 12, ureter. Orientation markers: A, anterior; L, lateral; M, medial; P, posterior

**Figure 2:**
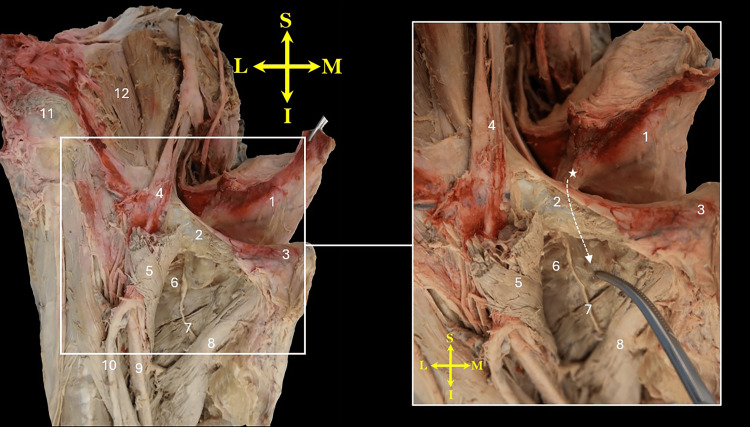
Anterior view of the right hemipelvis and upper thigh showing the bladder herniating through the obturator foramen into the upper medial thigh, positioned between the pectineus and external obturator muscles. The pectineus muscle is cut and reflected laterally. The neck of the hernia is marked by an asterisk (*), and its tip is held by a curved hemostat. Note the external portion of the hernia, primarily composed of fat packet, is flattened between the two muscles. The direction of the hernia passing under the superior pubic ramus is indicated by a curved dashed arrow. 1, bladder; 2, superior pubic ramus; 3, pubic tubercle; 4, external iliac vessels passing into the thigh; 5, reflected pectineus muscle; 6, external obturator muscle (obscured by fatty tissue); 7, obturator nerve 8, adductor longus muscle; 9, femoral vein; 10, greater saphenous vein; 11, anterior superior iliac spine; 12, iliacus muscle. Orientation markers: I, inferior; L, lateral; M, medial; S, superior

**Figure 3:**
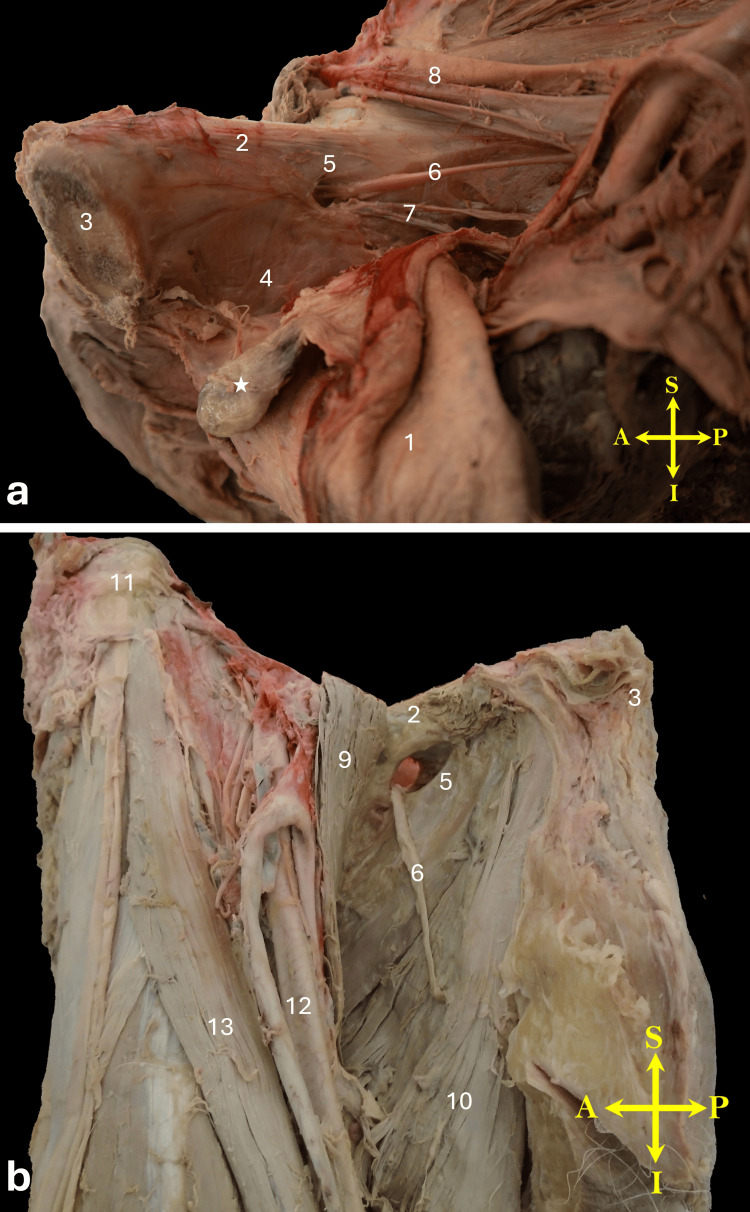
(a) Medial view of the right hemipelvis showing the bladder reflected downward and the obturator hernia reduced. Note the pedunculated bladder hernia marked with an asterisk (*), and the relatively enlarged obturator foramen from the internal view. (b) Anterior view of the upper thigh after reduction of the obturator hernia. The obturator foramen appears significantly enlarged on the external view. 1, bladder; 2, superior pubic ramus; 3, body of the pubis; 4, internal obturator muscle covered by its fascia; 5, obturator foramen; 6, obturator nerve; 7, obturator vessels; 8, external iliac vessels passing into the thigh; 9, reflected pectineus muscle; 10, adductor longus muscle; 11, anterior superior iliac spine; 12, femoral vein; 13, sartorius muscle. Orientation markers: A, anterior; I, inferior; L, lateral; M, medial; S, superior; P, posterior

**Figure 4:**
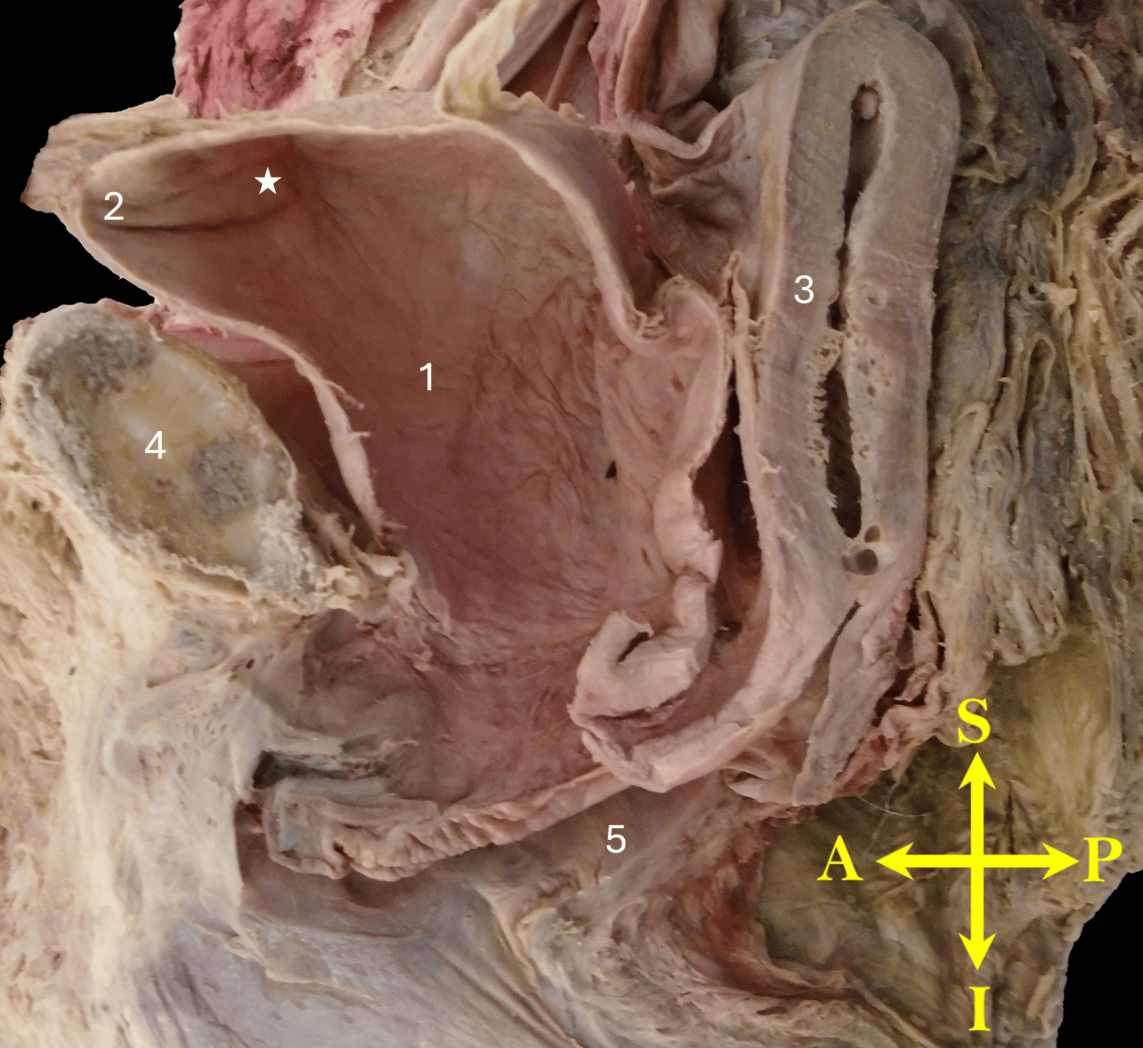
Midsagittal view of the right hemipelvis illustrating an enlarged urinary bladder. No other apparent pelvic or bladder pathology is observed. An asterisk (*) marks the internal region likely corresponding to the bladder hernia. 1, bladder; 2, apex of the bladder; 3, uterus; 4, pubis; 5, vaginal canal. Orientation markers: A, anterior; L, lateral; M, medial; P, posterior

The donor was an 80-year-old female, with a thin body habitus who had died of cancer. Her medical history was unavailable, aside from the recorded cause of death. During dissection, a bladder herniation was identified through the right obturator canal (Figure 1). The hernia originated from the anterolateral aspect of the upper bladder and extended externally between the external obturator and pectineus muscles (Figure 2). The hernia measured 5.2 cm in length and 2.6 cm in width at its neck, narrowing to 7 mm as it traversed the obturator canal. Reduction of the hernia was challenging due to fibrous attachments between the hernia sac and the wall of the obturator canal. After reduction, the right obturator canal was visibly dilated, measuring 20 × 9 mm externally (Figure 3). The hernia sac contained bladder wall tissue, fibrofatty elements, and a partial peritoneal covering. The bladder appeared moderately dilated (Figure 4), but no other abnormalities were observed. The left obturator canal appeared normal.

## Discussion

The findings of adhesions between the hernia sac and the obturator foramen, along with enlargement of the obturator foramen, in the present case likely indicate a chronic herniation. The pedunculated hernia sac, originating from the bladder near its dome, was grossly constricted at the level of the obturator foramen and externally compressed between the pectineus and external obturator muscles, further supporting the chronic nature of the herniation. We identified nine prior reports of obturator hernias involving the bladder or bladder diverticulum in both English and non-English literature (Table 1) [2,4-11].

**Table 1:** Previous cases of obturator bladder hernia. Abbreviations: CT, computed tomography; FDG PET, fludeoxyglucose-18 positron emission tomography scan; PET/CT; positron emission tomography-computed tomography; OF, obturator foramen. The reports of Bitker and Tasić are not included in this table. Source: Refs [2,4-11]

Case	Presentation	Risk Factors	Management
Gladstone (1901) [5]	Dissection findings	Elderly female	Not applicable
McCarthy (1976) [8]	Chronic back pain	No obvious risk factor (middle-aged man)	Pfannenstiel laparotomy with an extraperitoneal approach and suture closure of the OF
Fritz et al. (1997) [9]	Acute abdomen and hemorrhagic infarction of the bladder wall	Elderly female with massive fecal impaction and overdistension of the entire colon	Infrainguinal approach and pararectal lower abdominal laparotomy, partial resection of the superior pubic ramus, and reconstruction of the OF defect using mesh
Velásquez-López et al. (2008) [2]	Recurrent urinary tract infections and irritative voiding symptoms	Elderly female	Laparoscopic intraperitoneal repair with mesh placement
Kaneta et al. (2009) [6]	FDG PET and fused PET/CT images showing increased uptake at the OF	Elderly female with esophageal cancer, status post-chemoradiotherapy	Not reported
Kikkawa et al. (2009) [7]	Incidental findings on abdominal CT	Elderly female, frailty and history of breast cancer	Expectant management
Watanabe et al. (2016) [4]	Thigh pain and abdominal pain	Elderly female	Midline laparotomy, with the OF covered by a preperitoneal mesh fixed to Cooperʼs ligament

Notably, in 1901, Gladstone reported an incidental finding of a bladder obturator hernia and a contralateral obturator hernia involving the fallopian tube in an elderly woman during dissection [5]. Manifestations of obturator bladder hernias range from asymptomatic to chronic back pain, abdominopelvic or thigh pain, recurrent urinary tract infections, and acute abdomen. One previously reported clinical case was incidental finding managed expectantly [7]. When surgery is indicated, laparoscopic or open hernia repair, often with mesh reinforcement, is the preferred approach.

Obturator hernia can be a serious condition, commonly affecting elderly, frail women. Several risk factors contribute to its development [3,9,12,13]. Advanced age, female sex, and multiple pregnancies are associated with weakened pelvic muscles and connective tissues. Additionally, women tend to have a wider pelvis and a larger obturator canal. Thin or underweight individuals are at greater risk due to reduced protective adipose tissue around the obturator nerve and vessels. Conditions that increase intra-abdominal pressure, such as chronic constipation, chronic cough, or chronic obstructive pulmonary disease, further predispose individuals to this type of herniation. Prior surgeries or injuries to the pelvic region can weaken the area, making it more vulnerable to obturator herniation.

Obturator hernias are often unsuspected and frequently go undiagnosed. The classic presentation involves acute intestinal obstruction with bowel strangulation or incarceration, leading to significant morbidity and mortality due to delayed diagnosis and treatment. The reported incidence of obturator hernias is low. A 15-year retrospective study at the Mayo Clinic found that obturator hernias accounted for only 0.073% (11 out of 15,098) of all hernia repairs [14]. However, the actual incidence may be higher. In a retrospective analysis of laparoscopic extraperitoneal hernia repairs, obturator foramina was routinely explored in 293 patients undergoing repair of bilateral or recurrent inguinal hernias. Obturator hernias were identified in 20 cases, representing 6.82% of the patients [12]. This finding suggests that the true incidence of obturator hernia is likely higher than traditionally reported in the literature. The detection rate might increase further with routine pelvic imaging and if both men and women were equally evaluated [12].

Obturator hernias are classified into three types based on the extent of herniation [2,15]. In type I obturator hernias, preperitoneal fat and connective tissue enter the pelvic opening of the obturator canal. This type is often asymptomatic and commonly overlooked, usually causing only intermittent mild discomfort or pain. A type II obturator hernia is characterized by the dimpling of the peritoneum into the obturator canal, resulting in the formation of an empty peritoneal sac. This type typically presents with vague pelvic pain or discomfort, though symptoms can progress as the hernia worsens. Type III obturator hernias, the most severe form, involve the herniation of part of an organ into the obturator canal, resulting in a hernia that is not spontaneously reduced. Most cases of obturator hernias are discovered when they present as a type III hernia with small bowel obstruction [2,15,16].

## Conclusions

This case highlights the importance of recognizing chronic obturator hernias, particularly in frail, elderly patients who may present with nonspecific symptoms. While such hernias may go undetected until post-mortem or incidental findings, clinicians should consider them in patients with pelvic or groin discomfort, especially those with risk factors such as advanced age or a history of pelvic surgery. The findings suggest that obturator hernias may be more common than the current literature suggests, emphasizing the need for greater vigilance and routine assessment by healthcare providers.
